# In-Situ Raman Characterization of Initial Corrosion Behavior of Copper in Neutral 3.5% (wt.) NaCl Solution

**DOI:** 10.3390/ma12132164

**Published:** 2019-07-05

**Authors:** Ming Liu, Jun Li

**Affiliations:** 1 State Key Laboratory for Strength and Vibration of Mechanical Structures, Xi’an Jiaotong University, Xi’an 710049, China; 2 Corrosion and Protection Center, University of Science and Technology Beijing, Beijing 100083, China

**Keywords:** copper, Cl^−^ medium, initial corrosion behavior, in-situ Raman characterization

## Abstract

In order to investigate the role of chloride ion in the corrosion film formation of copper and its evolution over time, the initial corrosion behavior of copper in neutral 3.5% (wt.) NaCl solution was characterized by in-situ Raman spectroscopy along with electrochemical tests. The results demonstrated that the cuprous chloride complexes, such as CuCl and ${\mathrm{CuCl}}_{2}^{-}$ were produced through electrode processes, while the cuprite, ${\mathrm{Cu}}_{2}O$ seemed to be formed via the chemical precipitation reaction instead of a direct electrochemical transformation from the metal matrix or CuCl and it occurred rather slowly. At the open circuit potential, the chlorides were generated first in the initial 2 h and then they transformed to the oxides with the ${\mathrm{CuCl}}_{2}^{-}$ content in the interface increasing. The in-situ Raman characterization directly evidenced the previously reported mechanism of growth of oxide layers on copper surfaces in neutral ${\mathrm{Cl}}^{-}$ media and clearly showed the formation of a corrosion product film and its evolution over time. The electrochemical tests corresponded to the results of in-situ Raman characterization well.

## 1. Introduction

Copper has found broad application in industrial fields such as nuclear waste processing [1,2,3,4], printed circuit boards [5,6,7] and it is one of the more stable metals due to its high overpotential for hydrogen evolution and the absence of surface oxides in acidic solutions. However, in spite of its self-protective function, copper still undergoes serious deterioration in some highly aggressive environments. For instance, in an aerated ${\mathrm{Cl}}^{-}$ medium, the corrosion of copper occurs at a noticeable rate and it can change the performance of copper dramatically [8].

The corrosion of copper in ${\mathrm{Cl}}^{-}$ media has been widely studied and the results have indicated that ${\mathrm{Cl}}^{-}$ has a marked impact on the copper corrosion mechanism [9,10,11,12]. There have been three models proposed for the anodic dissolution of copper in ${\mathrm{Cl}}^{-}$ media in accordance with the following reactions [8]:

Case 1

(1) $$\mathrm{Cu}+{2\mathrm{Cl}}^{-}↔{\mathrm{CuCl}}_{2}^{-}+e^{-}$$

Case 2

(2) $$\mathrm{Cu}↔{\mathrm{Cu}}^{+}+e^{-}$$

(3) $${\mathrm{Cu}}^{+}+{2\mathrm{Cl}}^{-}↔{\mathrm{CuCl}}_{2}^{-}$$

Case 3

(4) $$\mathrm{Cu}+{\mathrm{Cl}}^{-}↔\mathrm{CuCl}+e^{-}$$

(5) $$\mathrm{CuCl}+{\mathrm{Cl}}^{-}↔{\mathrm{CuCl}}_{2}^{-}$$

Cases 1 and 3 present the direct formation of cuprous chloride complexes from the metal while Case 2 involves the dissolution of copper as ${\mathrm{Cu}}^{+}$ in the first instance. The above reactions are usually supposed to be reversible and under charge transfer and mass transport mixed control close to the corrosion potential. The ${\mathrm{CuCl}}_{2}^{-}$ can be coordinated further when the ${\mathrm{Cl}}^{-}$ concentration gets greater than 1 M as following reactions [13]:

(6) $${\mathrm{CuCl}}_{2}^{-}+{\mathrm{Cl}}^{-}↔{\mathrm{CuCl}}_{3}^{2-}$$

(7) $${\mathrm{CuCl}}_{3}^{2-}+{\mathrm{Cl}}^{-}↔{\mathrm{CuCl}}_{4}^{3-}$$

In seawater or NaCl electrolytes with a ${\mathrm{Cl}}^{-}$ concentration close to 0.55 M, ${\mathrm{CuCl}}_{2}^{-}$ is thought to be the main cuprous chloride complex.

The three possible cases given in reactions (1)–(5) seem to be independent of pH because there is no involvement of $H^{+}$ or ${\mathrm{OH}}^{-}$. However, it is well known that the electrode reactions are interface processes and they can be affected by adsorbates on electrode surfaces, such as ${\mathrm{OH}}^{-}$. In addition, the resultants, cuprous chloride complexes, may be transformed to other species such as oxides in alkaline or neutral media further. Therefore, it is necessary that it be exploited thoroughly. In-situ characterization techniques help to reveal the reactions between copper and ${\mathrm{Cl}}^{-}$ remarkably well. Suggs and Bard [14,15] studied the dissolution of copper in acidic aqueous ${\mathrm{Cl}}^{-}$ solutions with in-situ electrochemical scanning tunneling microscope (STM). They found that a ${\mathrm{Cl}}^{-}$ adlattice with a ($\sqrt{2}\times \sqrt{2}$) *R*$45^{o}$ structure grows on Cu (100) electrode in 10 mM hydrochloric acid (pH 2.1) and this structure can be maintained even at higher dissolution potentials. Stage edges along the {100} directions will be preferentially etched because of high density of kinks and the open structure of the underlying copper edges. The adsorbed ${\mathrm{Cl}}^{-}$ weakens the bonds between the topmost copper layer and bulk copper, especially at kink sites, and the open structure is the favored formation of the ${\mathrm{CuCl}}_{2}^{-}$ configuration furthermore. The authors proposed that ${\mathrm{Cl}}^{-}$ reacts with a copper atom at the kink site forming a cuprous chloride species as in reaction (4) and it dissolves from the edge to form a ${\mathrm{CuCl}}_{2}^{-}$ with another ${\mathrm{Cl}}^{-}$. Analogously, a ${\mathrm{Cl}}^{-}$ adlattice with a ($6\sqrt{3}\times 6\sqrt{3}$) *R*$30^{o}$ structure can also be formed on a Cu (111) electrode with step edges as the preferred dissolution sites. In alkaline solutions, the effects of ${\mathrm{Cl}}^{-}$ on the initial stage of anodic dissolution of copper get much more complicated because of the involvement of ${\mathrm{OH}}^{-}$ and the formation of oxide layers as a result. Kunze et al. [16] studied the mechanism of growth of passive layer on Cu (111) in NaOH solution (pH 11–13) with in situ STM as well and found that it varies with the concentration ratio of ${\mathrm{Cl}}^{-}$ to ${\mathrm{OH}}^{-}$. Atomically-resolved images demonstrated that in the underpotential range of oxidation, the topmost Cu (111) planes are reconstructed into a less densely packed hexagonal structure and ${\mathrm{OH}}^{-}$ are adsorbed in the threefold hollow sites of this structure to form a precursor surface which adopts the structure of (111) oriented ${\mathrm{Cu}}_{2}O$ in the presence of ${\mathrm{Cl}}^{-}$. At lower concentrations, no influences of ${\mathrm{Cl}}^{-}$ were observed on the growth of the ${\mathrm{OH}}^{-}$ adlattice. With ${\mathrm{Cl}}^{-}$ content increasing, it competes with the ${\mathrm{OH}}^{-}$ for adsorption at preferential sites, i.e., step edges, where copper atoms are mobile. At higher ${\mathrm{Cl}}^{-}$ concentration, the preferential adsorption of ${\mathrm{OH}}^{-}$ at the step edges of the copper surface is fully blocked by the formation of a non-ordered copper chloride compound, but the preferential adsorption still dominates the center of terraces. The presence of ${\mathrm{Cl}}^{-}$ does not significantly modify the structural characteristics of the ${\mathrm{Cu}}_{2}O$ layer formed at sufficiently positive potentials but it enhances localized cupper dissolution at the oxide step edges and makes the step edges of oxide layer rougher and indented.

In neutral ${\mathrm{Cl}}^{-}$ media, it may be difficult to form the oxide precursor through the reaction of ${\mathrm{OH}}^{-}$ like that in alkaline solutions, but the adsorption of ${\mathrm{OH}}^{-}$ or oxygen cannot be eliminated totally. As reported by by Suggs and Bard [15], in a neutral KCl solution (10 mM, pH 6.5) STM images revealed the same ${\mathrm{Cl}}^{-}$ adlattice structure on Cu (111) as that observed in 10 mM hydrochloric acid. However, there were many point defects present in KCl solution. The authors ascribed the presence of these point defects to impurities from the solution or oxygen in the ${\mathrm{Cl}}^{-}$ adlattice. In addition, ${\mathrm{Cu}}_{2}O$ can exist stably in the neutral media according to the Pourbaix diagrams for the Cu/$H_{2}O$/${\mathrm{Cl}}^{-}$ system [17] but its growth lacks direct in-situ characterization in spite of amounts of electrochemical tests. Therefore, in this work, the initial corrosion behavior of copper in neutral 3.5% (wt.) NaCl solution was characterized by in-situ Raman spectroscopy along with electrochemical tests and the formation of the corrosion product film and its evolution over time were demonstrated clearly.

## 2. Experimental

### 2.1. Medium and Electrodes

All reagents used in the present research were provided by Sinopharm Chemical Reagent Co., Ltd. and they are analytical grade. The medium in the tests was 3.5% (wt.) NaCl solution with initial oxygen concentration of 7.43 ppm and pH 6.3 at room temperature that was prepared with commercial reagent and deionized water. The purity of copper plate used in this work was more than 99.99 wt%. Copper electrodes were embedded in epoxy resin with a working area of 10 mm × 10 mm exposed for electrochemical tests and sealed in teflon with a cross-section of Φ5 mm exposed for in-situ Raman characterization. Prior to testing, the working surface was grounded with carbide abrasive papers gradually to #7000, degreased with ethanol, rinsed with deionized water, blow-dried in compressed air and immersed immediately in test medium.

### 2.2. Electrochemical Tests

Electrochemical tests were conducted within a classical three-electrode cell open to air under stagnant conditions in an ambient environment with a pretreated copper electrode as the working electrode, a saturated calomel electrode (SCE) as the reference electrode and a platinum sheet as the auxiliary electrodes, respectively. All potentials below were vs. SCE. The measurements were carried out with Princeton VersaSTAT 3F. The open circuit potential (OCP) of the copper electrode in NaCl solution during the 1st h was recorded. During the 12 h immersion, electrochemical impedance spectra (EIS) were measured at OCP from 100 kHz to 10 mHz with four points per decade under the excitation of a sinusoidal wave of 5 mV amplitude. The potentiodynamic polarization tests were measured from −0.25 vs. OCP to 0.2 V vs. SCE with a step size of 0.5 mV $s^{-1}$ after the copper electrode had been immersed in medium for at least 30 min to be stationary. All tests were conducted at least three times.

### 2.3. In-Situ Raman Characterization

The in-situ Raman characterization was performed in a special cell with an aforementioned prepared copper electrode facing up as working electrode, a platinum ring around the copper electrode as the auxiliary electrode and a saturated Ag/AgCl electrode being the reference electrode, which has a potential of 40 mV vs. SCE. All measured potential values were translated into vs. SCE. The electrodes were equipped in a teflon container full of NaCl solution with a quartz window placed over the copper electrode to avoid contamination. The Raman spectra were obtained with a Renishaw Raman spectrograph with the spectral resolution of 2 ${\mathrm{cm}}^{-1}$ and wave number reproducibility of 0.2 ${\mathrm{cm}}^{-1}$. A He-Ne laser of 785 nm was used as the incident radiation. All measurements were conducted at about 5 mW. The potential-dependent Raman spectra were measured by changing the potential of copper electrode from −1.2 to 0.6 V and then reversing to −1.2 V with a step size of 0.2 V per 3 min that was long enough for the system reaching a steady state. The real-time Raman spectra for copper corroding in NaCl solution were recorded once per 5 min during the initial 2 h and per 2 h from the 2nd to 12th h. In the case of long term immersing, solution in the cell was replaced periodically through circulating conduits. The potential-dependent spectra were obtained with laser focusing on the same location considering that the whole electrode surface could be supposed to be homogeneous with its potential being controlled. However, the real-time spectra were measured with the focus location varying every time in order to reflect the overall evolution and make the results statistically significant. What should be point is that the spectrograph has a depth of field of 2 μm so that it not only observes the electrode surface but also detects the solution at the interface. All tests were conducted at least three times. The obtained Raman spectra were processed with LabSpec software.

## 3. Results and Discussion

### 3.1. Potential-Dependent Raman Spectra

**Figure 1:**
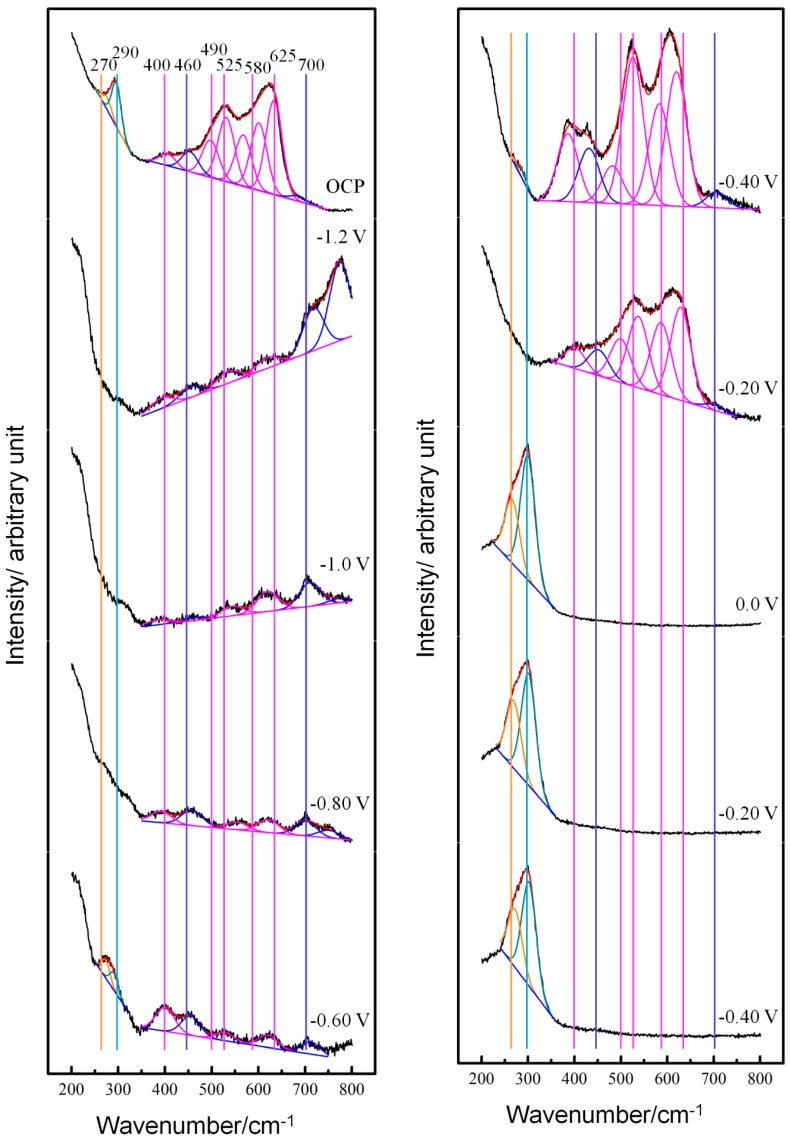

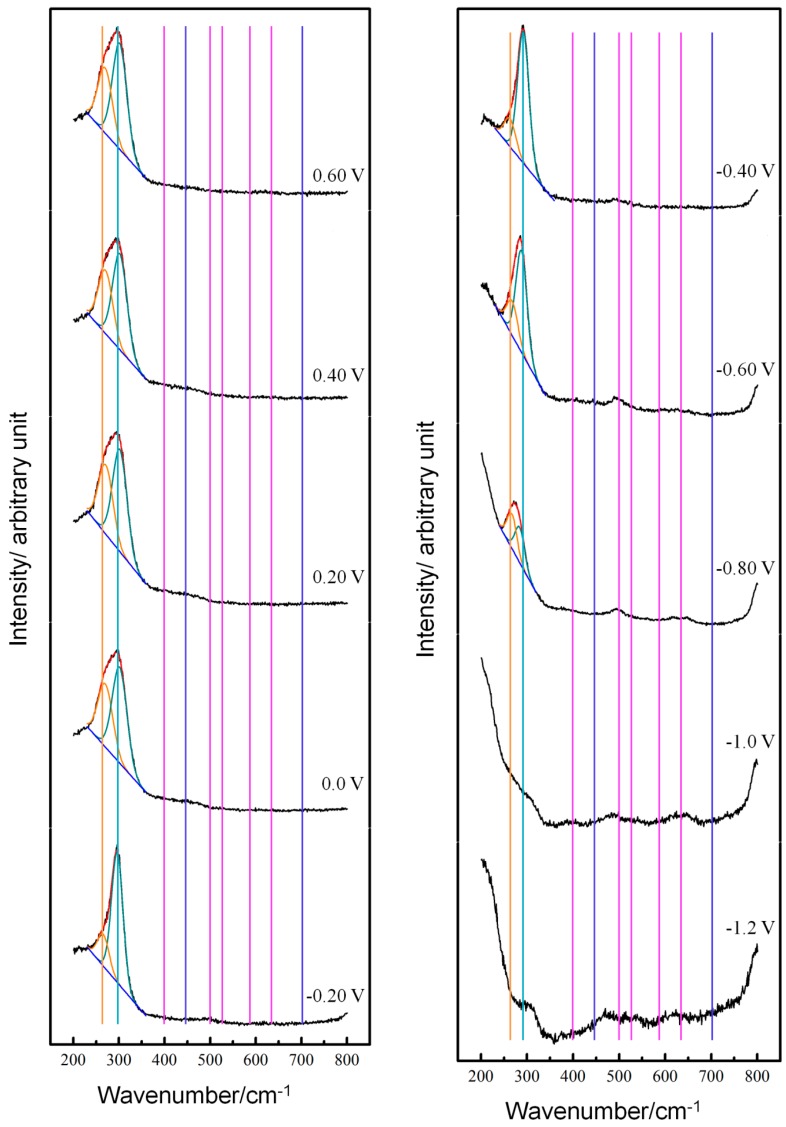
In-situ Raman spectra and fitted Gaussian components from copper electrode surface in neutral 3.5% (wt.) NaCl solution at open circuit potential (OCP) and with applied potential varying from −1.2 to 0.60 V and then reversing to −1.2 V.

The original in-situ Raman spectra and fitted Gaussian components from the copper electrode surface in neutral NaCl solution under different applied potential are shown in Figure 1. In general, there appear two main features in these spectra. The narrow band located in the region of 250–320 ${\mathrm{cm}}^{-1}$ can be ascribed to Cu-Cl vibration (chlorides) [18,19,20] and the broad band in the frequency range from 350 to 750 ${\mathrm{cm}}^{-1}$ arises from Cu-O and Cu-OH vibrations (oxides) [21,22,23,24,25,26,27,28,29], respectively. More specifically, the presence of the major ~625 ${\mathrm{cm}}^{-1}$ peak together with the shoulder at ~525 ${\mathrm{cm}}^{-1}$ is consistent with the formation of ${\mathrm{Cu}}_{2}O$. The minor peak around 400 ${\mathrm{cm}}^{-1}$ could be taken as the characteristic feature that ${\mathrm{Cu}}_{2}O$ is generated. Analysis based on group theory demonstrated that the broad peak around 625 ${\mathrm{cm}}^{-1}$ is infrared active and its appearance in the Raman spectra is most likely associated with disorder in ${\mathrm{Cu}}_{2}O$ surface film which breaks the symmetry of the lattice [25]. The Gaussian components around 460, 580 and 700 ${\mathrm{cm}}^{-1}$ may arise from ${\mathrm{Cu}(\mathrm{OH})}_{2}$, ${\mathrm{Cu}}_{2}{\mathrm{Cl}(\mathrm{OH})}_{3}$, ${\mathrm{Cu}(\mathrm{OH},\;\mathrm{Cl})}_{2}\cdot {2H}_{2}O$, and so on [24,26,27]. The narrow band in the frequency range from 250 to 320 ${\mathrm{cm}}^{-1}$ can be decomposed into two Gaussian components located around 270 and 290 ${\mathrm{cm}}^{-1}$, respectively [18,19]. The low-frequency one can be ascribed to absorbed ${\mathrm{Cl}}^{-}$ or CuCl film, and the other one is due to ${\mathrm{CuCl}}_{2}^{-}$. The characteristic peaks of CuO are not observed in experimental Raman spectra, especially those around 350 ${\mathrm{cm}}^{-1}$ [25,30,31].

As shown in Figure 1, the Raman features at OCP arose from the oxides generated during the preparation of electrode and adsorbed ${\mathrm{Cl}}^{-}$. When the electrode potential was polarized to −1.2 V, the oxides and chlorides were reduced so their feature bands diminished. Their features emerged again with the electrode potential shifting positively to −0.6 to −0.4 V because of the adsorption of ${\mathrm{Cl}}^{-}$ and ${\mathrm{OH}}^{-}$ in the underpotential range of oxidation. When the electrode potential was polarized more positive than 0 V, the features of the chlorides got dramatically stronger so that they overwhelmed the characteristics of the adsorbed ${\mathrm{OH}}^{-}$. The features of the chlorides kept pronounced until the electrode potential reversed to −0.8 V, by which point the chlorides had been largely reduced.

**Figure 2:**
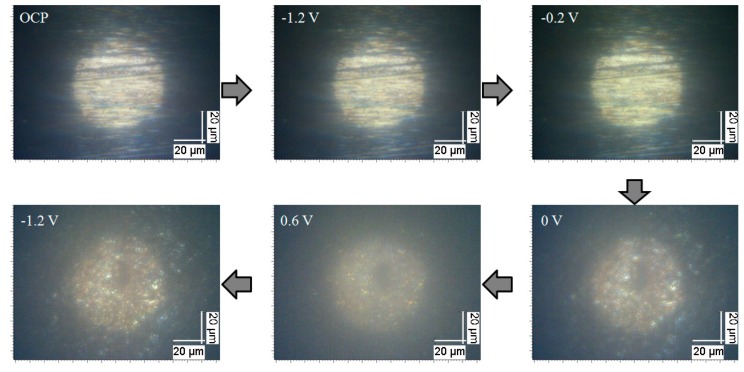
Snapshots of the copper surface in neutral 3.5% (wt.) NaCl solution at OCP and with applied potential varying from −1.2 to 0.60 V and then reversing to −1.2 V, which shows that a porous film was formed on copper surface with the electrode potential polarized to about 0 V and it was reduced after the potential reversed to −1.2 V.

The variation of Raman spectra with applied potential demonstrates that the formation and reduction of chlorides are electrode processes and they are potential-dependent. Anodic polarization accelerates the generation of CuCl film and ${\mathrm{CuCl}}_{2}^{-}$. When the electrode potential reversed to ~−0.20 V, the CuCl film adhered to copper surface was reduced first so that its Raman feature diminished accordingly. Then, with the electrode potential cathodically polarized further, the ${\mathrm{CuCl}}_{2}^{-}$ in the interface between the copper and solution were also reduced, as indicated by their characteristics peak. This indicates that the above-mentioned Cases 1 and 2 may not be applicable in the current case and an insoluble CuCl film can be formed besides the ${\mathrm{CuCl}}_{2}^{-}$, which can be verified by the snapshots obtained at different potentials in Figure 2. In addition, Yuan et al. [32,33] have proved evidently that at the initial stage, the ${\mathrm{Cu}}^{+}$ ions are formed first and they react to produce the CuCl and/or cuprous chloride complexes further in 0.5 M NaCl solution with the digital holography. Therefore, there may be equilibrium among the copper base, ${\mathrm{Cu}}^{+}$, CuCl and other cuprous chloride complexes, as follows:

$$\mathrm{Cu}↔{\mathrm{Cu}}^{+}+e^{-}$$

(8) $${\mathrm{Cu}}^{+}+{\mathrm{Cl}}^{-}↔\mathrm{CuCl}$$

$${\mathrm{Cu}+\mathrm{Cl}}^{-}↔\mathrm{CuCl}+e^{-}$$

$${\mathrm{CuCl}+\mathrm{Cl}}^{-}↔{\mathrm{CuCl}}_{2}^{-}$$

The ${\mathrm{Cu}}_{2}O$ is commonly supposed to be formed via the precipitation reaction [34]:

(9) $${2\mathrm{CuCl}}_{2}^{-}+2{\mathrm{OH}}^{-}↔{\mathrm{Cu}}_{2}{O\;+\;H}_{2}O+\;4{\mathrm{Cl}}^{-}$$

in the presence of ${\mathrm{CuCl}}_{2}^{-}$ instead of a direct chemical or electrochemical transformation from the metal matrix or CuCl and its thermodynamics equilibrium versus CuCl does not depend on the electrode potential but relies on the pH and ${\mathrm{Cl}}^{-}$ concentration according to the Pourbaix diagram of Cu/$H_{2}O$/${\mathrm{Cl}}^{-}$ system [17]. At a pH value above 5, ${\mathrm{Cu}}_{2}O$ rather than CuCl should be formed with the following equilibrium shifting to the right:

(10) $${2\mathrm{CuCl}\;+\;2\mathrm{OH}}^{-}↔{\mathrm{Cu}}_{2}{O\;+\;H}_{2}O+\;2{\mathrm{Cl}}^{-}$$

which comes from summing reactions (5) and (9). However, the above potential-dependent Raman spectra indicate that precipitation reaction (9) occurred rather slowly so that the features of oxides were overwhelmed by the characteristics of chlorides with electrode reactions (2,4,5,8) accelerated by anodic polarization and amounts of chlorides generated.

**Figure 3:**
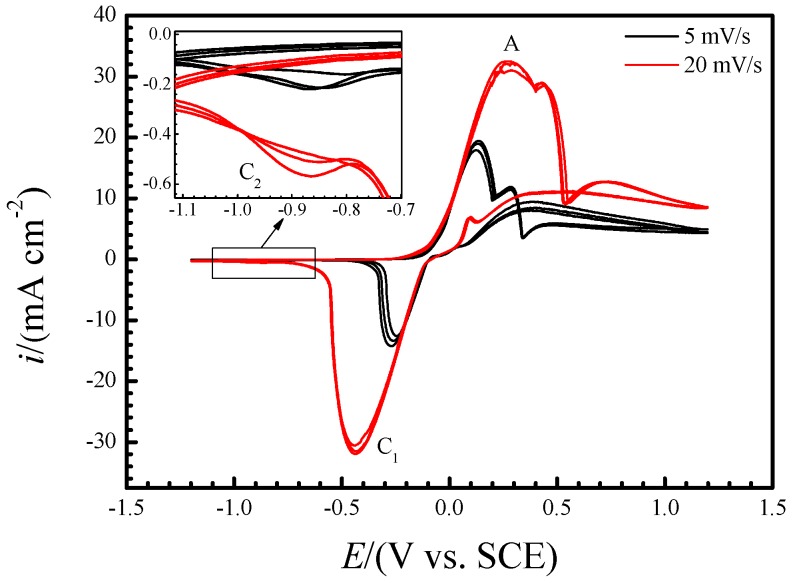
Stationary cyclic voltammograms after at least three cycles of copper electrode in neutral 3.5% (wt.) NaCl solution at room temperature.

Figure 3 shows the stationary cyclic voltammograms after at least three cycles of copper electrode in neutral 3.5% (wt.) NaCl solution at room temperature and it evidences above interpretation of Raman spectra, too. According to previous studies, the oxidation peak A at ~−0.25 V is caused by the formation of cuprous chloride complexes (CuCl and ${\mathrm{CuCl}}_{2}^{-}$) and it represents a shoulder peak because of the precipitation of CuCl film on electrode surface [35]. The cathodic peak C_1_ corresponds to their reduction. As shown in Figure 3, the peak current densities of A and C_1_ and the difference between their peak potentials increased with scan rate increasing, which is the characteristic of reactant diffusion in the solution and also evidences that these two peaks are related to formation and reduction of CuCl film and ${\mathrm{CuCl}}_{2}^{-}$ as well. Besides the C_1_, there exists another cathodic peak at ~−0.9 V (C_2_), as shown in the inserted enlarged view in the Figure 3, which is due to the reduction of ${\mathrm{Cu}}_{2}O$ [36,37]. Compared with that of C_1_, the peak current density of C_2_ is much smaller which consists with the above interpretation of Raman characterization that the precipitation of ${\mathrm{Cu}}_{2}O$ occurs pretty slowly and it can be hardly accelerated by the anodic polarization, either.

### 3.2. Real-Time Raman Spectra at OCP

**Figure 4:**
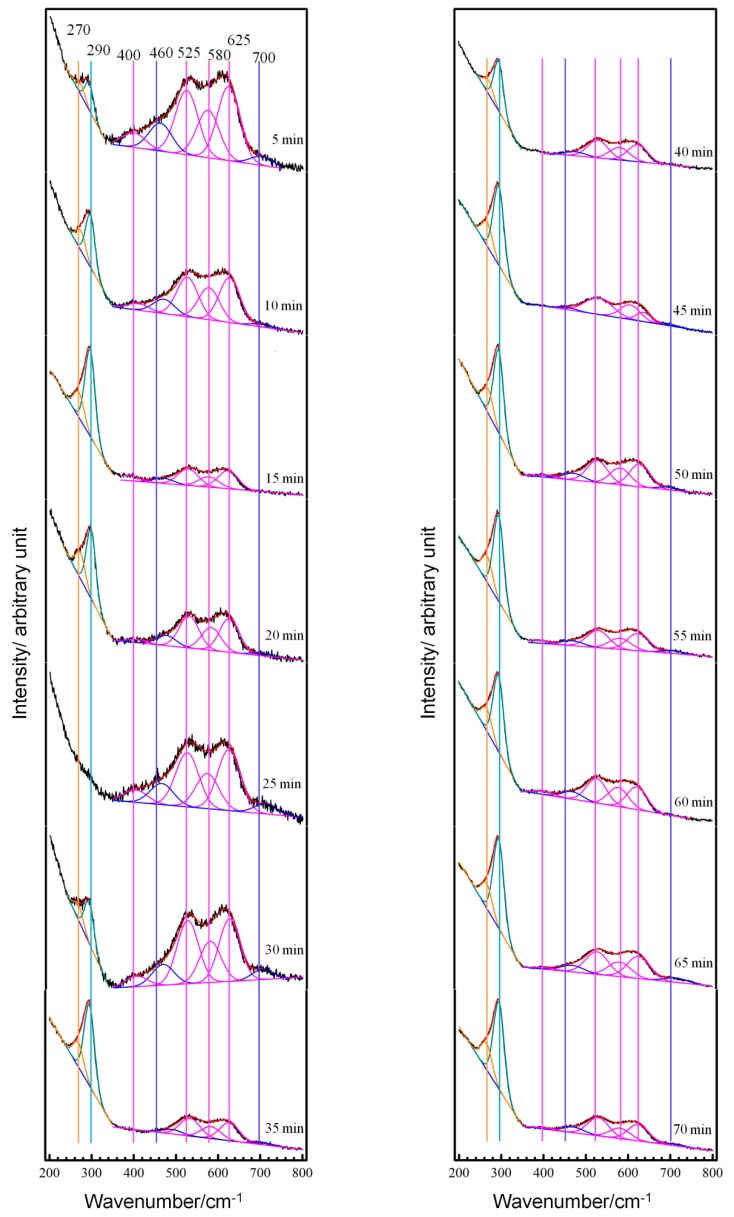

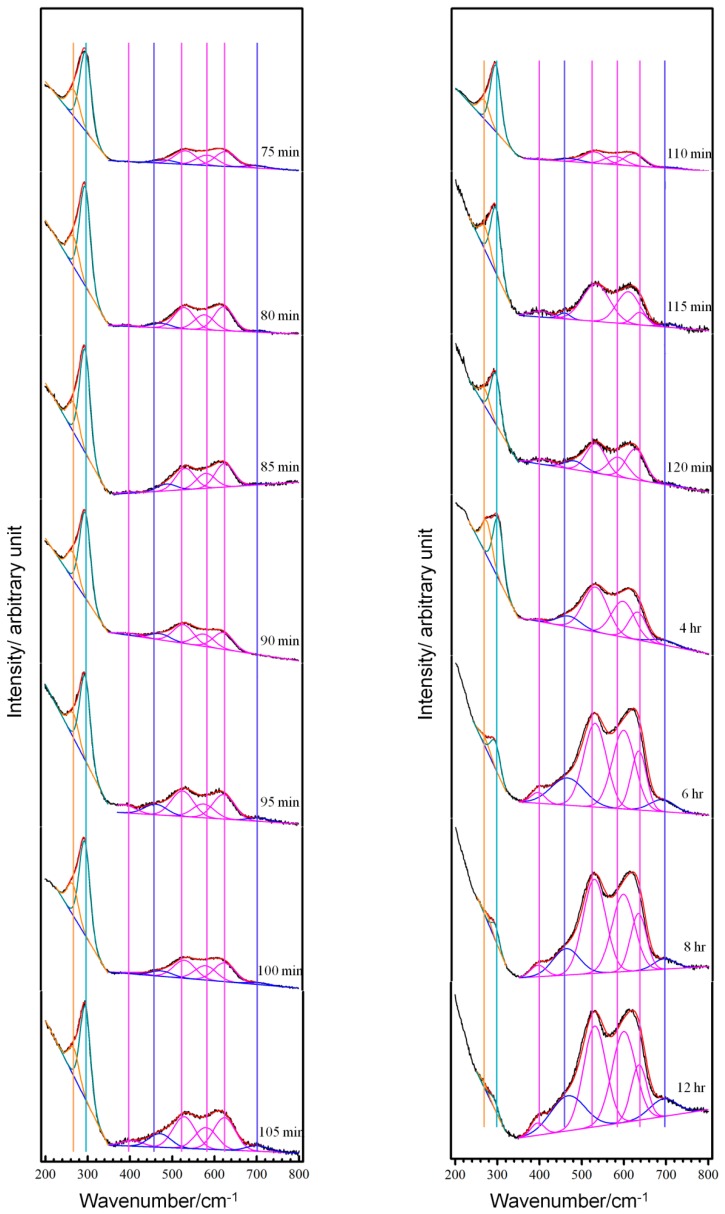
In-situ Raman spectra and fitted Gaussian components from copper electrode surface in neutral 3.5% (wt.) NaCl solution at OCP with immersion times varying from 0 to 12 h.

The original in-situ Raman spectra and fitted Gaussian components from the copper electrode surface in NaCl solution during the initial 12 h are shown in Figure 4. When copper was initially immersed into the electrolyte, its Raman spectrum the exhibited weak characteristic peaks of Cu-Cl vibration arising from adsorbed ${\mathrm{Cl}}^{-}$ on a copper surface as shown in the graph of 5 min from Figure 4. The relatively stronger band of Cu-O and Cu-OH vibrations is due to oxides resulting from the preparation of specimen in air. Then, CuCl and ${\mathrm{CuCl}}_{2}^{-}$ were formed and their characteristic peaks got more pronounced during immersion from 5 to 15 min. The spectra of 25 and 30 min showed pronounced features of oxides, because at those moments the laser was focus somewhere the ${\mathrm{OH}}^{-}$ are preferentially adsorbed, such as on the terraces. However, chlorides were generated universally during this period and their features got more and more pronounced with the immersion time going up to 60 min. During the 2nd h of immersion, the experimental spectra changed very little and the features of the chlorides remained much stronger than those of oxides until an immersion time approaching 110 min. After an immersion time beyond 110 min, the features of CuCl and ${\mathrm{CuCl}}_{2}^{-}$ diminished and the characteristic band of oxides got increasingly pronounced with immersion times extending from 2 to 12 h, as shown in Figure 4.

As demonstrated with the above potential-dependent Raman spectra, the formations of chlorides are electrode processes and they react much more rapidly than the precipitation of ${\mathrm{Cu}}_{2}O$. That is, ${\mathrm{Cu}}_{2}O$ is more thermodynamically stable at the corrosion potential of the copper electrode in the present test electrolyte, but kinetics favors the formation of chlorides first. This is why the Raman features of CuCl and ${\mathrm{CuCl}}_{2}^{-}$ got pronounced at the beginning of immersion and kept strong until the immersion time approaching 110 min. Chlorides were formed universally on the copper surface. Meanwhile, oxides could also be precipitated slightly when local energy and concentration fluctuations were enough to overcome the adverse conditions from kinetics elsewhere. The former prevailed over the latter and amounts of chlorides were formed during the initial 2nd h. With CuCl precipitating and its film thickening to a certain extent, the transfer of ${\mathrm{Cl}}^{-}$ and ${\mathrm{CuCl}}_{2}^{-}$ through holes inside the film was hindered and the formation of chlorides was inhibited largely. On the contrary, formations of oxides were accelerated with the concentration of ${\mathrm{CuCl}}_{2}^{-}$ in the interface between the copper and solution increasing. Namely, the CuCl film transformed to ${\mathrm{Cu}}_{2}O$ gradually and the Raman features of oxides got increasingly pronounced after immersion for 2 h, as shown in Figure 4.

**Figure 5:**
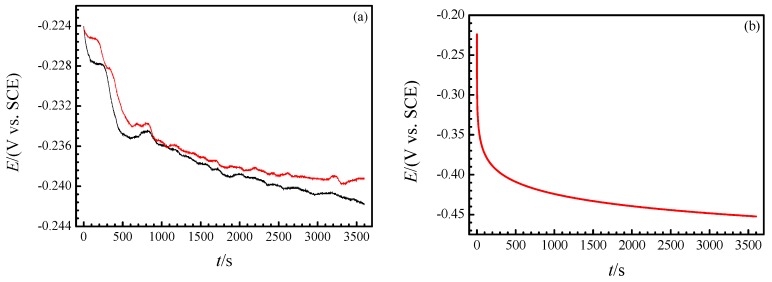
Experimental OCP diagram of copper electrode in neutral 3.5% (wt.) NaCl solution at room temperature during the 1st h (**a**) and corresponding theoretical one (**b**).

Figure 5a shows the OCP diagram of the copper electrode in NaCl solution during the 1st h and it can be interpreted by the real-time Raman spectra, too. As shown, the OCP shifted negatively with the immersion time going on and tended to be stable eventually. In the meantime, some fluctuations occurred discontinuously, and the plot has a stair-step shape in the initial stage. The theoretical OCP is deduced on the assumption that only copper anodic dissolution occurs but its reverse reaction and reduction of oxygen are not considered, as is shown in Figure 5b. The interface between metal and the solution is modeled with a plane-parallel capacitor and it follows the equation below:

(11) $$\frac{\mathrm{dE}}{\mathrm{dt}}=-\frac{i}{C}$$

in which i is charge current that equals to the dissolution current of copper to the solution, E represents the electrode potential and C is double layer capacity. Videlicet, dissolution of ${\mathrm{Cu}}^{+}$ to solution is regarded as a charge process of which power is not from impressed voltage but from the degradation of Gibbs free energy for the whole electrode system. On the other hand, i is a function of E:

(12) i = f(E)

Combine Equations (11) and (12) and following equation can be obtained:

(13) $$-C\frac{\mathrm{dE}}{\mathrm{dt}}=f\left(E\right)$$

Transform and resolve Equation (13) and a relationship between E and immersing time, t, can be obtained as following:

(14) $$t\;=\;-C\int_{\varphi_{0}}^{E}\frac{1}{f(\mu )}d\mu$$

in which C is assumed to be constant with respect to E, $\varphi_{0}$ is the electrode potential the moment copper is just immersed in the test electrolyte (t = 0) and it may be considered as the potential of zero charge assuming that the characteristic adsorption of water molecules and ${\mathrm{Cl}}^{-}$ is fast enough.

**Figure 6:**
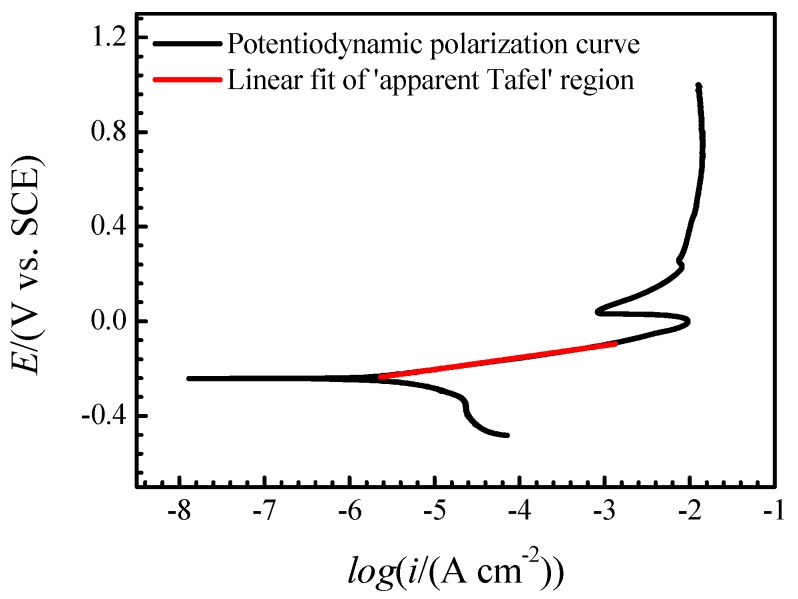
Potentiodynamic polarization curve of the copper electrode in neutral 3.5% (wt.) NaCl solution at room temperature and the linear fit of the ‘apparent Tafel’ region.

Previous studies [8] reported that the dissolution of copper in ${\mathrm{Cl}}^{-}$ medium shows ‘apparent Tafel’ behavior, which is thought to be under the control of mixed charge transfer and mass transport processes in the region next to corrosion potential. This is evidenced by the potentiodynamic polarization curve in Figure 6 and the following fitting function between E and i can be obtained in the ‘apparent Tafel’ region:

(15) E/V = 0.05065log(i/A) + 0.04971

i.e.,

(16) $${f(E)\;=\;10}^{\frac{E-0.04971}{0.05065}}$$

Set C as 20 μF ${\mathrm{cm}}^{-2}$ and take it in Equation (14) along with (16) and the OCP of copper in NaCl solution can be resolved as a function of immersion time, t, as following:

(17) $$E/V\;=\;0.04971-0.05065\log(\frac{10^{6}t/s}{0.4399}+2.535\times 10^{5})$$

as shown in Figure 5b.

The experimental OCP of copper electrode immersed in the test electrolyte during the 1st h presents a trend similar to the theoretical trend and it conforms to the in-situ Raman characterization well. First, the continuous dissolution of ${\mathrm{Cu}}^{+}$ into solution makes the electrode potential shift negatively all along. Next, the formation of chlorides is easier and faster but it has no effect of depolarization. Finally, adsorption of ${\mathrm{OH}}^{-}$ and precipitation of ${\mathrm{Cu}}_{2}O$ along with oxygen depolarization reaction are thermodynamically beneficial but slower and occasionally they result in a stair-step shape and fluctuations in the OCP plot of the copper electrode in the test electrolyte, as shown in Figure 5a. It should be noted that the stable potential of experimental OCP is much more positive than that of the theoretical, which is a result of the reverse reaction of copper dissolution and in particular, reduction of oxygen.

**Figure 7:**
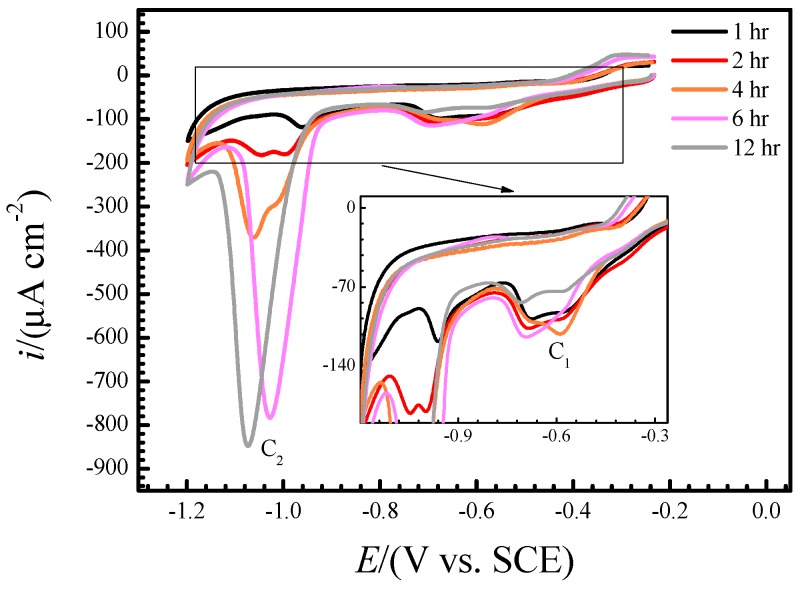
Voltammograms that swept from the OCP to 1.2 V and then reversed to OCP of copper electrode in NaCl solution at room temperature after it had been immersed for 1, 2, 4, 6 and 12 h.

As demonstrated with the above real−time Raman spectra, the precipitation of oxides was accelerated after immersion for ~2 h with the concentration of ${\mathrm{CuCl}}_{2}^{-}$ in the interface between copper and solution increasing, which can be confirmed by the corresponding voltammograms, too. Figure 7 shows the voltammograms that swept from the OCP to −1.2 V and then reversed to OCP of copper electrode in NaCl solution at room temperature after it had been immersed for 1, 2, 4, 6 and 12 h. Similar to the stationary cyclic voltammograms in Figure 3, the cathodic sweeping curves in Figure 7 also present two reduction peaks (C_1_ and C_2_) at about −0.6 and −1 V, respectively, and both of them include more than one sub−peak. As demonstrated above, the peak C_1_ can be ascribed to the reduction of CuCl film and ${\mathrm{CuCl}}_{2}^{-}$ while C_2_ is due to the reduction of ${\mathrm{Cu}}_{2}O$. Figure 7 indicates that ${\mathrm{Cu}}_{2}O$ accumulated during immersion but the amounts of CuCl and ${\mathrm{CuCl}}_{2}^{-}$ remained small all along. This may be because the latter transformed to ${\mathrm{Cu}}_{2}O$ and most of ${\mathrm{CuCl}}_{2}^{-}$ diffused into bulk solution in the meantime.

**Figure 8:**
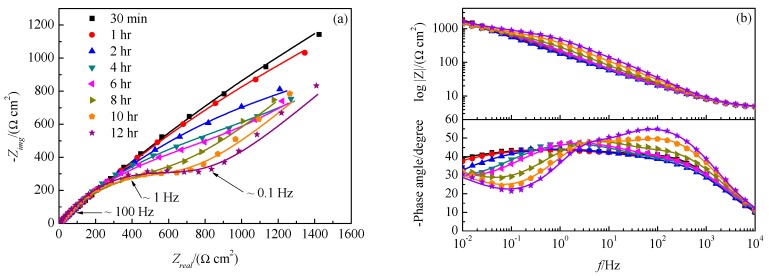
Nyquist (**a**) and Bode (**b**) impedance plots of copper electrode in neutral 3.5% (wt.) NaCl solution at room temperature after immersion for 0.5, 1, 2, 4, 6, 8, 10 and 12 h.

**Figure 9:**
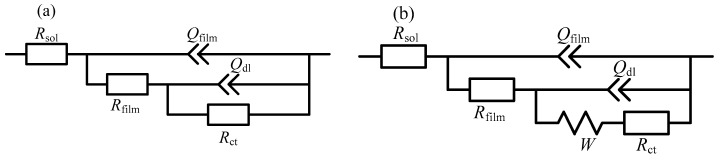
Equivalent electrical circuits used to fit electrochemical impedance spectra (EISs) in Figure 8: (**a**) for 30 min, 1 h, 2 h and (**b**) for 2, 4, 6, 8, 10 and 12 h.

The EIS can also reflect the evolution of corrosion product film during immersing for the initial 12 h. Figure 8 shows the Nyquist (a) and Bode (b) plots of copper electrode in NaCl solution after immersing for 0.5, 1, 2, 4, 6, 8, 10 and 12 h. In the first 2 h, the EISs showed two capacitive loops. After then, the characteristic frequency of the capacitive loop in the lower-frequency range increased and from the 4th h on, the Warburg impedance appeared because of the consumption of oxygen. Equivalent electrical circuits (EECs) [38,39] shown in Figure 9 were utilized to fit EISs in Figure 8, in which *R* represents resistance, *Q* for the constant phase element (CPE) and *W* the Warburg impedance for the diffusion process. The impedance of CPE is difined as

(18) $$Z_{\mathrm{CPE}}={\left[Q\;{\left(j\omega \right)}^{n}\right]}^{-1}$$

The parameter *Q* and *n* are independent of the frequency. The value of *n* could be 0 ≤ *n* ≤ 1 or −1. A value of 1 describes an ideal capacitor, 0 describes an ideal resistance and −1 an inductor. Values of *n* differing from 1 or 0 could be attributed to in-depth, lateral or time distributions of the observed phenomenon (the dispersion effect), and when *n* = 0.5 the CPE represents a Warburg impedance, i.e.,

(19) $$Z_{W}=W^{-1}{\left(j\omega \right)}^{-0.5}$$

$Q_{\mathrm{film}}$ and $R_{\mathrm{film}}$ are associated with the corrosion product film. $Q_{\mathrm{dl}}$ represents double-layer capacitance and $R_{\mathrm{ct}}$ is charge transfer resistance. The calculated parameters for equivalent circuit components are shown in Table 1. As shown, $R_{\mathrm{film}}$ barely changed during the initial 2 h but it increased dramatically from then to the 12th h, which was caused by the transformation of CuCl film to ${\mathrm{Cu}}_{2}O$ precipitates. On contrary, $R_{\mathrm{ct}}$ decreased during the immersing due to that CuCl film is insulative but ${\mathrm{Cu}}_{2}O$ precipitates are semiconductor. ${\mathrm{Cu}}_{2}O$ precipitating on copper surface can support electrode reactions, especially the cathodic oxygen consumption, which consequently reduced the interfacial charge transfer resistance. This is consistent with the in-situ Raman characterization well.

## 4. Conclusions

In this work, the in-situ Raman spectra characterized the initial corrosion behavior of copper in neutral 3.5% (wt.) NaCl solution clearly. The potential-dependent spectra demonstrated that the formations of chlorides such as CuCl and ${\mathrm{CuCl}}_{2}^{-}$ are electrode processes and they can be accelerated by anodic polarization, while the ${\mathrm{Cu}}_{2}O$ is verified to be formed via the chemical precipitation reaction instead of a direct electrochemical transformation from the metal matrix or CuCl and it occurs rather slowly. Correspondingly, the real-time spectra at OCP shows that the ${\mathrm{Cl}}^{-}$ plays an important role in the corrosion process of copper in ${\mathrm{Cl}}^{-}$ media and chlorides are generated first in the initial 2 h, and then the formations of oxides are accelerated with the concentration of ${\mathrm{CuCl}}_{2}^{-}$ in the interface between copper and solution increasing to some extent. Consequently, the CuCl film transforms to ${\mathrm{Cu}}_{2}O$ gradually. Electrochemical tests can specifically evidence the above results of in-situ Raman characterization.

## Figures and Tables

**Table 1:** Calculated parameters for equivalent circuit components in Figure 9.

Immersion Time	*R*_sol_/ $\left({\Omega \;\mathrm{cm}}^{2}\right)$	*Q*_film_/ $\left(\Omega^{-1}\;{\mathrm{cm}}^{-2}\;s^{n}\right)$	*n*_film_	*R*_film_/ $\left({\Omega \;\mathrm{cm}}^{2}\right)$	*Q*_dl_/ $\left(\Omega^{-1}\;{\mathrm{cm}}^{-2}\;s^{n}\right)$	*n*_dl_	*R*_ct_/ $\left({\Omega \;\mathrm{cm}}^{2}\right)$	*W*/ $\left(\Omega^{-1}\;{\mathrm{cm}}^{-2}\;s^{0.5}\right)$
30 min	4.5	3.4 × 10^−4^	0.72	27.4	1.7 × 10^−3^	0.48	1.46 × 10^4^	
1 h	4.4	4.0 × 10^−4^	0.70	29.4	1.8 × 10^−3^	0.48	1.03 × 10^4^	
2 h	4.3	4.6 × 10^−4^	0.69	23.8	1.7 × 10^−3^	0.49	4577	
4 h	4.2	5.1 × 10^−4^	0.67	35.7	1.0 × 10^−3^	0.57	1622	4.8 × 10^−3^
6 h	4.0	6.5 × 10^−4^	0.65	53.8	6.9 × 10^−4^	0.61	1209	4.4 × 10^−3^
8 h	4.2	4.5 × 10^−4^	0.69	65.8	4.2 × 10^−4^	0.68	667.4	3.9 × 10^−3^
10 h	4.3	3.4 × 10^−4^	0.71	179.1	2.0 × 10^−4^	0.84	529	3.9 × 10^−3^
12 h	4.4	2.6 × 10^−4^	0.73	373.3	1.2 × 10^−4^	0.99	338.4	3.7 × 10^−3^
